# 883 Extubation Failure in the Burn Unit

**DOI:** 10.1093/jbcr/iraf019.414

**Published:** 2025-04-01

**Authors:** Charles Voigt, Colette Galet, Ryan Frede, Alexander Kurjatko

**Affiliations:** University of Iowa Health Care Burn Center; University of Iowa Health Care Burn Center; University of Iowa Health Care Burn Center; University of Iowa Health Care Burn Center

## Abstract

**Introduction:**

The decision to extubate a patient from the ventilator is challenged by the concern for post-extubation respiratory failure. While there is no set benchmark for extubation failure rates, reported rates in burn units range from 12.3-30%. Prior studies suggest that extubation failure is associated with increased mortality, hospital length of stay (LOS), and pneumonia. Our institution developed a ventilator liberation protocol involving daily evaluation of ventilator settings and patient characteristics, followed by a spontaneous breathing trial. This study was performed to evaluate the effectiveness of our extubation protocol and to identify areas of potential improvement.

**Methods:**

This is a retrospective cohort study. Patients admitted to our burn unit from 7/10/2015 to 6/30/2023 who had been intubated for over 24 hours were identified in our burn registry. Patients who self-extubated, had tracheostomy, died prior to extubation, or were palliatively extubated were excluded. Demographics, comorbidities, injuries, and hospital course were collected. Data were analyzed using Fisher’s exact test and non-parametric Mann-Whitney U test to assess differences between extubation failure and success. P < 0.05 was considered significant.

**Results:**

Ninety-three patients were included. Eight (8.6%) were identified as extubation failure, defined as requiring reintubation for any reason within 72 hours of extubation. There were no significant differences in total body surface area burned, presence of inhalation injury, hospital length of stay, or mortality between groups. Patients identified as extubation failure were significantly older, (65 vs. 50 years, p = 0.037), more likely to present with a history of heart failure (37.5% vs. 4.7%, p = 0.013) and diabetes (50% vs. 14.1%, p = 0.028). They also had higher rates of ventilator associated pneumonia (37.5% vs. 5.9%, p = 0.019) and were more likely to stay longer on a ventilator (9 vs. 4 days, p = 0.007). There was a non-significant trend towards increased blood urea nitrogen, glucose, and phosphorous, as well as decreased eye Glasgow Coma Scale score in those who failed extubation. There was no difference in rate of compliance to any steps in our ventilator liberation protocol between groups.

**Conclusions:**

This study highlights the effectiveness of our institution’s ventilator liberation protocol. Extubation failure was 8.6% during the study period, lower than previously reported in the burn literature. Patients who experienced an extubation failure were more likely to develop ventilator associated pneumonia and stayed longer on a ventilator.

**Applicability of Research to Practice:**

Opportunity to improve the protocol exists by identifying older, diabetic, and heart failure patients as they were more likely to experience an extubation failure.

**Funding for the Study:**

N/A

